# Pharmaceutical Cocrystals of Ethenzamide: Molecular Structure Analysis Based on Vibrational Spectra and DFT Calculations

**DOI:** 10.3390/ijms23158550

**Published:** 2022-08-01

**Authors:** Mei Wan, Jiyuan Fang, Jiadan Xue, Jianjun Liu, Jianyuan Qin, Zhi Hong, Jiusheng Li, Yong Du

**Affiliations:** 1 Centre for THz Research, China Jiliang University, Hangzhou 310018, China; wm1315286544@163.com (M.W.); jiyuanfang@foxmail.com (J.F.); jianjun@cjlu.edu.cn (J.L.); jyqin@cjlu.edu.cn (J.Q.); hongzhi@cjlu.edu.cn (Z.H.); lijsh@cjlu.edu.cn (J.L.); 2 Department of Chemistry, Zhejiang Sci-Tech University, Hangzhou 310018, China; jenniexue@126.com

**Keywords:** ethenzamide, hydroxybenzoic acids, pharmaceutical cocrystal, terahertz time domain spectroscopy, Raman spectroscopy, density functional theory

## Abstract

Pharmaceutical cocrystals can offer another advanced strategy for drug preparation and development and can facilitate improvements to the physicochemical properties of active pharmaceutical ingredients (APIs) without altering their chemical structures and corresponding pharmacological activities. Therefore, cocrystals show a great deal of potential in the development and research of drugs. In this work, pharmaceutical cocrystals of ethenzamide (ETZ) with 2,6-dihydroxybenzoic acid (26DHBA), 2,4-dihydroxybenzoic acid (24DHBA) and gallic acid (GA) were synthesized by the solvent evaporation method. In order to gain a deeper understanding of the structural changes after ETZ cocrystallization, terahertz time domain spectroscopy (THz-TDS) and Raman spectroscopy were used to characterize the single starting samples, corresponding physical mixtures and the cocrystals. In addition, the possible molecular structures of ETZ-GA, ETZ-26DHBA and ETZ-24DHBA cocrystals were optimized by density functional theory (DFT). The results of THz and Raman spectra with the DFT simulations for the three cocrystals revealed that the ETZ-GA cocrystal formed an O−H∙∙∙O hydrogen bond between the -OH of GA and oxygen of the amide group of the ETZ molecule, and it was also found that ETZ formed a dimer through a supramolecular amide–amide homosynthon; meanwhile, the ETZ-26DHBA cocrystal was formed by a powerful supramolecular acid–amide heterosynthon, and the ETZ-24DHBA cocrystal formed the O−H∙∙∙O hydrogen bond between the 4-hydroxy group of 24DHBA and oxygen of the amide group of the ETZ molecule. It could be seen that in the molecular structure analysis of the three cocrystals, the position and number of hydroxyl groups in the coformers play an essential role in guiding the formation of specific supramolecular synthons.

## 1. Introduction

“Pharmaceutical cocrystal” refers to the molecular compounds that exhibit solid-state APIs and one or more cocrystal coformers (CCFs) at room temperature, in which the molecular compounds can be formed through a variety of noncovalent interactions, including a hydrogen bond, π–π stacking, halogen bond, van der Waals force interactions and others [1,2,3,4,5,6]. In recent decades, due to the rise of pharmaceutical cocrystal technology, researchers have shifted from showing solicitude for the polymorphs, solvent compounds and salts to the study of APIs of cocrystals [7]. Cocrystals can effectively improve the physicochemical properties of related APIs, such as melting point, stability, bioavailability and solubility. At the same time, the chemical structure, inherent biological activity and corresponding pharmacological activity of APIs are only slightly affected in most cases [8,9]. Therefore, research on pharmaceutical cocrystals has been increasingly pursued in the crystal pharmaceutical industry and gradually applied to the research of anti-inflammatory and analgesic drugs, demonstrating a positive impact on the improvement of the physicochemical properties of anti-inflammatory and analgesic drugs.

**Figure 1:**
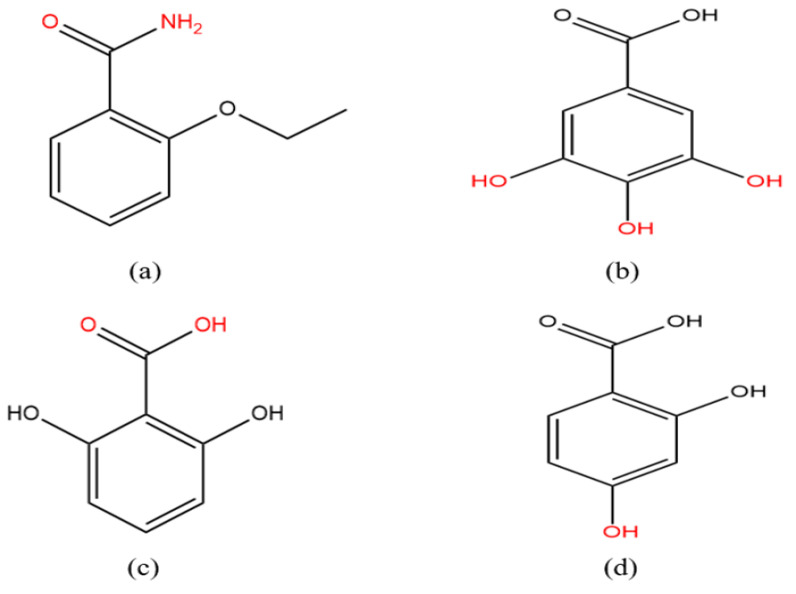
Molecular structures of (**a**) ETZ, (**b**) GA, (**c**) 26DHBA and (**d**) 24DHBA (the red shows the functional groups involved in the intermolecular hydrogen bond interaction between ETZ and various coformers in this work).

In this work, pharmaceutical cocrystals of ethenzamide (ETZ, molecular structure as shown in Figure 1) were synthesized by the crystal engineering method. ETZ is a nonsteroidal anti-inflammatory drug (NSAID) with analgesic, antipyretic, anti-inflammatory and anti-rheumatic effects [10,11,12]. It is mainly combined with paracetamol, aspirin, dipyrone, allyl isopropyl acetyl urea, caffeine and ibuprofen [13,14]. ETZ drugs encounter significant problems because of their low solubility and bioavailability. NSAIDs can be used as antioxidants, in which oxidants will reduce the pharmacological effect of NSAIDs by an anti-inflammatory mechanism. Therefore, the CCFs added to the ETZ formulation are antioxidants, whereby the ETZ activity can usually be improved. For the above reasons, in the present study, we selected gallic acid monohydrate (GA, antibacterial agent, an antioxidant), 2,6-dihydroxybenzoic acid (26DHBA, γ-resorcylic acid, an antioxidant) and 2,4-dihydroxybenzoic acid (24DHBA, β-resorcylic acid, an antioxidant and anti-inflammatory agent) as coformers, with the molecular structures shown in Figure 1.

In the past two decades, Moribe et al. [15] have reported a cocrystal of ETZ and thiourea. According to powder X-ray diffraction (PXRD), differential scanning calorimetry (DSC), thermogravimetric analysis (TGA) and hot stage microscopy (HSM), researchers have reported a variety of ETZ cocrystals [16,17,18,19,20]. Przybylek et al. [21] have reported cocrystals of ETZ and different benzoic acids. Kozak et al. [22] evaluated the effect of grinding time for ETZ and glutaric acid cocrystal formation according to characterization techniques such as solid-state nuclear magnetic resonance (SSNMR) and Fourier transform infrared spectroscopy (FT-IR). They also have reported cocrystals of ETZ and three aliphatic dicarboxylic acids by SSNMR, FT-IR, DSC and single-crystal X-ray diffraction (SCXRD) [23]. A range of analytical techniques, such as SSNMR, DSC, TGA, SCXRD, PXRD and FT-IR, have been applied to study various ETZ cocrystals. However, there are few reports on the vibrational spectra at the molecular level, such as THz and Raman spectra. THz spectroscopy offers solid-state crystal molecular structures with a clear fingerprint, which may be used to distinguish and analyze the subtle structural changes caused by intermolecular interactions effectively [24,25,26]. Different from THz spectroscopy, involving an absorption effect, Raman spectroscopy involving light scattering can offer us abundant structural information on particular molecules, such as the vibrational transition and electronic polarization of molecules, as well as the vibration of groups in different molecules [26,27,28,29]. Therefore, THz and Raman spectra are complementary: combining the detection results of THz and Raman spectra, we can completely describe the low-frequency vibration of different pharmaceutical cocrystals [30,31].

In this work, single starting samples, corresponding physical mixtures and the corresponding cocrystals were studied by using vibrational spectroscopy technologies, such as THz and Raman spectroscopy. Moreover, DFT was applied to optimize the possible molecular structures of ETZ-GA, ETZ-26DHBA and ETZ-24DHBA cocrystals for molecular structure analysis and vibrational mode attribution [32,33]. Among the three cocrystal molecular structures, both the position and number of hydroxyl groups in the above three coformers have an important influence on the formation of specific supramolecular synthons, which provides a reference for the expansion of novel solid-state ETZ with improved physicochemical properties.

## 2. Materials and Methods

### 2.1. Material and Sample Preparation

Solid-state ETZ (purity 98%) was purchased from J&K Chemical Company (Shanghai, China). The samples of GA (purity 98%), 26DHBA (purity 98%) and 24DHBA (purity > 98%) were obtained from Sigma-Aldrich Company (Shanghai, China). Methanol and ethanol were purchased from Hangzhou Gaojing Fine Chemical Company. Acetonitrile was purchased from Tianjin Kemio Chemical Reagent Company. All chemicals were used without further purification.

ETZ-GA cocrystal: ETZ (200 mg, 1.21 mmol) and GA (113.82 mg, 0.605 mmol) were taken in a 2:1 stoichiometric ratio, placed in a glass evaporating dish containing 10 mL of ethanol. The mixed solution was heated to 70 °C to dissolve the two materials, and allowed to crystallize by slow evaporation of the solvent at room temperature. Colorless block crystals were obtained after 4 days.

ETZ-26DHBA cocrystal: ETZ (165.19 mg, 1 mmol) and 26DHBA (154.12 mg, 1 mmol) were taken in a 1:1 stoichiometric ratio, placed in a glass evaporating dish containing 10 mL of methanol. The mixed solution was heated to 80 °C to dissolve the two materials, and allowed to crystallize by slow evaporation of the solvent at room temperature. Light brown block crystals were obtained after 3 days.

ETZ-24DHBA cocrystal: ETZ (165.19 mg, 1 mmol) and 24DHBA (154.12 mg, 1 mmol) were taken in a 1:1 stoichiometric ratio, placed in a glass evaporating dish containing 10 mL of acetonitrile. The mixed solution was heated to 80 °C to dissolve the two materials, and allowed to crystallize by slow evaporation of the solvent at room temperature. Colorless block crystals were obtained after 3 days.

The physical mixtures of ETZ and three CCFs (GA, 26DHBA and 24DHBA) were obtained by placing the corresponding two starting components in a conical centrifuge tube, followed by spinning on a vortex mixer for 2 min. The mass and stoichiometric ratio of the two components involved in the physical mixture were consistent with their cocrystals.

ETZ, three CCFs, corresponding physical mixtures and the cocrystals were ground into powder and placed into a tablet press with a pressure of 6 MPa for approximately 1 min. Tableting was performed for THz detection. The sample after tableting was a small disc with a thickness of 2 mm and a diameter of 13 mm, and it was stored hermetically. For the detection of the Raman spectrum, only a small amount of powder sample was needed, without further sample preparation.

### 2.2. THz/Raman Instrument and Procedures

The THz spectra of ETZ, three CCFs, corresponding physical mixtures and the cocrystals were measured by the THz TDS Z2 system (Zomega Co., New York, NY, USA). This standard device, based on photoconductive switches, is used to characterize the THz transmission spectra of target materials. THz radiation was achieved by the Ti: Sapphire oscillator ultrafast laser pulse system (Spectral Physics, Owen, CA, USA) with an optical switch and optical pulse serial driver detection, in which the frequency is 75 MHz, the pulse width is 100 fs, and the center wavelength is 780 nm. A high-resistivity silicon lens and a parabolic mirror were used to collimate the THz radiation. The samples were measured under dry nitrogen at room temperature. The samples were placed in a THz-TDS system, which used high-purity nitrogen purging to decrease environmental moisture absorption by radiation. Each sample needed to be measured three times, and then the THz spectrum of each sample was finally obtained by averaging the three THz spectra of the sample. The data of each sample and reference sample (no sample rack) were recorded in the time domain of the THz electric field, in which the frequency response of the sample was divided by the frequency response of the reference sample through the process of fast Fourier transform (FFT) to obtain the THz absorption spectrum. The Raman vibrational spectrum was obtained by using a Fourier transform Raman spectrometer (Thermo Nicolet Corporation, Madison, WI, USA) and a semiconductor pumped solid-state laser (wavelength 1064 nm) as a near-infrared source. In the wavenumber range of 150–3500 cm^−1^, more than 500 scans were performed, with a spectral resolution of 2 cm^−1^ and a laser working power of 150 mW.

### 2.3. DFT Theoretical Calculations

**Figure 2:**
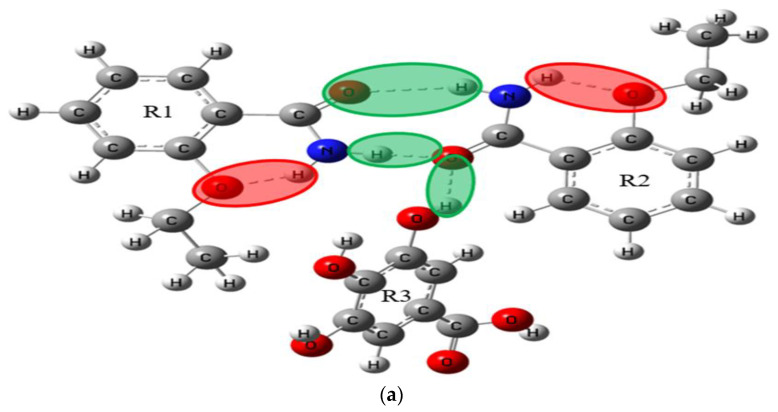

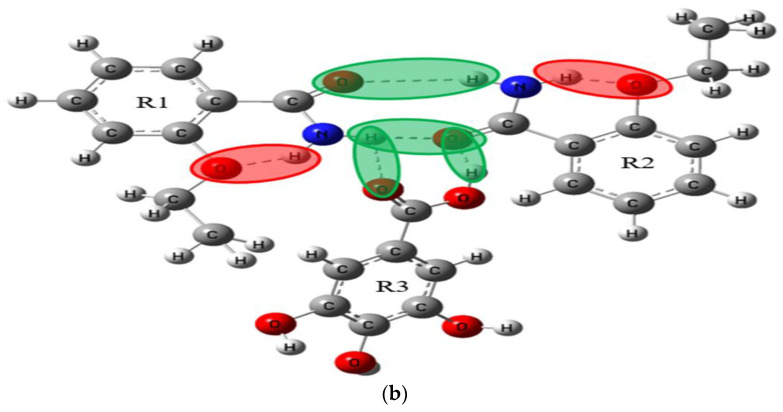
Schematic diagram of molecular structures for possible theoretical ETZ-GA cocrystal (**a**) form I and (**b**) form II.

**Figure 3:**
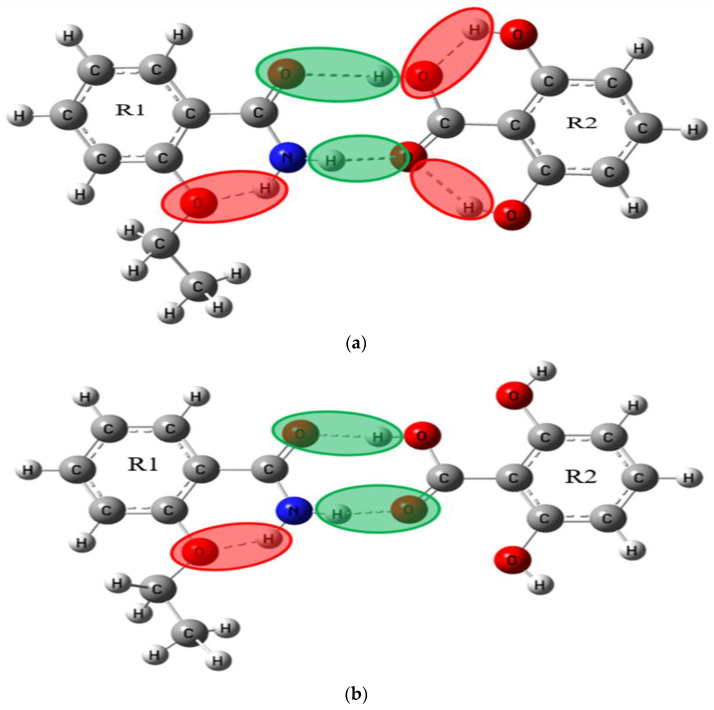
Schematic diagram of molecular structures for possible theoretical ETZ-26DHBA cocrystal (**a**) form I and (**b**) form II.

**Figure 4:**
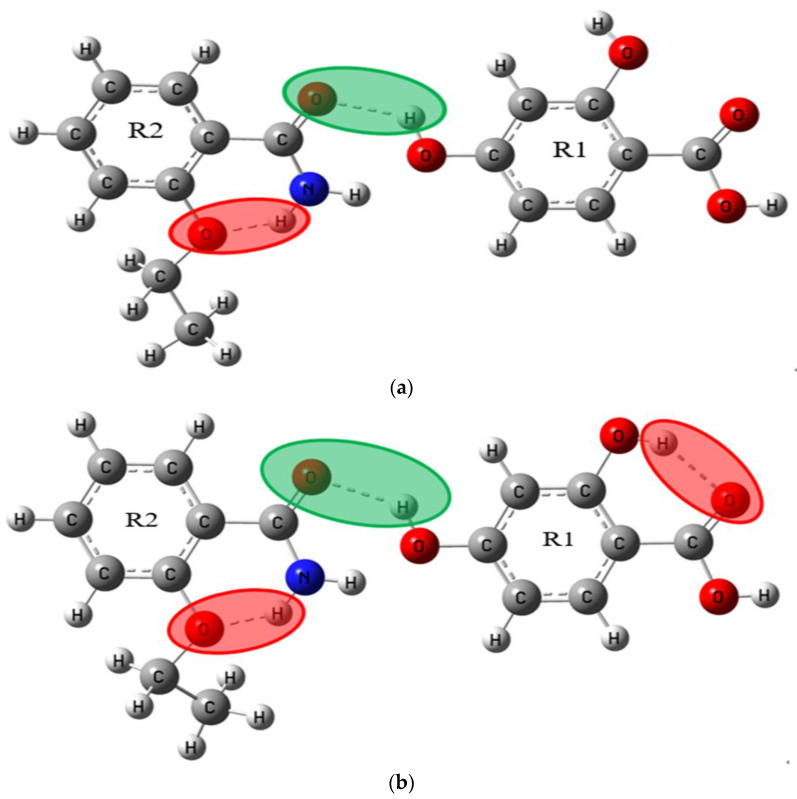
Schematic diagram of molecular structures for possible theoretical ETZ-24DHBA cocrystal (**a**) form I and (**b**) form II.

As a powerful tool for quantum chemical simulation, Gaussian software can simulate various geometric structures and spectral properties. In this paper, the theoretical structures of ETZ-GA, ETZ-26DHBA and ETZ-24DHBA cocrystals were simulated and optimized at the DFT level by using the B3LYP functional and 6-311++G (d, p) basis set in combination with Gaussian 09 and Gaussian-View software [34,35]. According to the structural information of ETZ and the three CCFs and the synthetic law of hydrogen bonds, we simulated two possible molecular structures in the theory of ETZ-GA, ETZ-26DHBA and ETZ-24DHBA cocrystals. In the figures, we show the intramolecular hydrogen bond interaction of ETZ with a red shadow, and the intermolecular hydrogen bond interaction between ETZ and the three coformers with a green shadow, as in Figure 2, Figure 3 and Figure 4.

Trivedi et al. [36] reported a cocrystal of ETZ and GA according to the characterization techniques of SCXRD, PXRD, FT-IR and DSC. Sarma et al. [37] reported three cocrystal polymorphs of ETZ and 24DHBA according to PXRD, FT-IR, Raman spectroscopy and DSC. They also reported a cocrystal of ETZ and 26DHBA according to SCXRD, PXRD, FT-IR, DSC and TGA characterization technologies [38]. However, the cocrystal structure analysis based on THz and Raman spectroscopy can provide more detailed information for observing the structural changes and vibrational modes of materials. In order to focus on describing the primary hydrogen bond synthon displayed in the molecular structure, the results of THz and Raman spectra were combined with DFT simulations, which have important implications for a better understanding of the structural features and vibrational modes of the three cocrystals. In the DFT calculations at B3LYP level, we used the full-width half maximum (FWHM) of 4.0 cm^−1^ to convolute the Lorentz line into the calculated vibrational modes.

## 3. Results and Discussion

### 3.1. THz Spectral Characterization and Analysis of ETZ-GA, ETZ-26DHBA and ETZ-24DHBA Cocrystals

**Figure 5:**
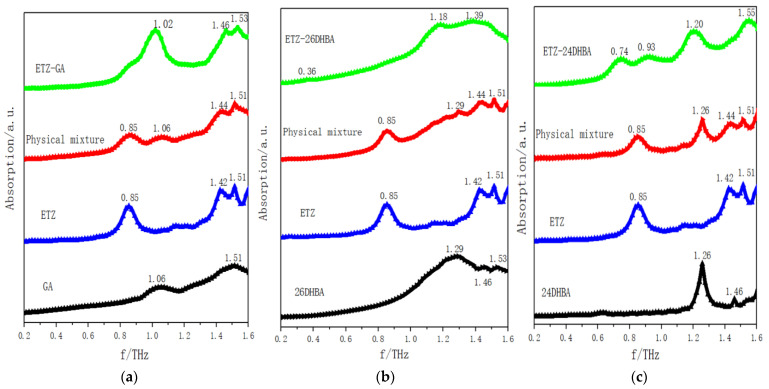
The THz spectra of (**a**) ETZ-GA cocrystal with ETZ, GA and physical mixture; (**b**) ETZ-26DHBA cocrystal with ETZ, 26DHBA and physical mixture; and (**c**) ETZ-24DHBA cocrystal with ETZ, 24DHBA and physical mixture in 0.2–1.6 THz spectral region.

THz spectroscopy, which can be used to study various molecular structures and intermolecular interactions, is a powerful tool to acquire low-frequency vibrational information on crystalline compounds [39]. The THz spectra of the single starting samples, corresponding physical mixtures and the cocrystals are shown in Figure 5. As shown in Figure 5a, there are four obvious characteristic peaks in the physical mixture at 0.85, 1.06, 1.44 and 1.51 THz, among which its characteristic peaks can be regarded as the linear superposition of the individual characteristic peaks of the two starting components (ETZ and GA). Because there are no intramolecular and intermolecular interactions during the simple mixing processes, there are three characteristic peaks in the ETZ-GA cocrystal at 1.02, 1.46 and 1.53 THz. However, the two starting components and their physical mixture show completely different characteristic peaks from the ETZ-GA cocrystal, which confirms the formation of the ETZ-GA cocrystal. Similarly, as shown in Figure 5b, there are three characteristic peaks in the ETZ-26DHBA cocrystal at 0.36, 1.18 and 1.39 THz. The two starting components and their physical mixture show completely different characteristic peaks from the ETZ-26DHBA cocrystal, which confirms the formation of the ETZ-26DHBA cocrystal. As shown in Figure 5c, there are four characteristic peaks in the ETZ-24DHBA cocrystal at 0.74, 0.93, 1.20 and 1.55 THz. The two starting components and their physical mixture show completely different characteristic peaks from the ETZ-24DHBA cocrystal, which can also confirm the formation of the ETZ-24DHBA cocrystal. As mentioned above, under specific experimental conditions, there will be noncovalent bond interactions between different components, such as hydrogen bonds, π–π accumulation, halogen bonds, van der Waals forces, etc., which can guide the characteristic cocrystal formation. Therefore, the cocrystal and the two starting components show completely different characteristic peaks, indicating that the starting components successfully form the corresponding cocrystal through noncovalent bond interaction, especially the driving force of a strong intermolecular hydrogen bond, which also verifies the reliability of the THz spectrum to provide solid-state crystal molecular structures with a clear fingerprint.

**Figure 6:**
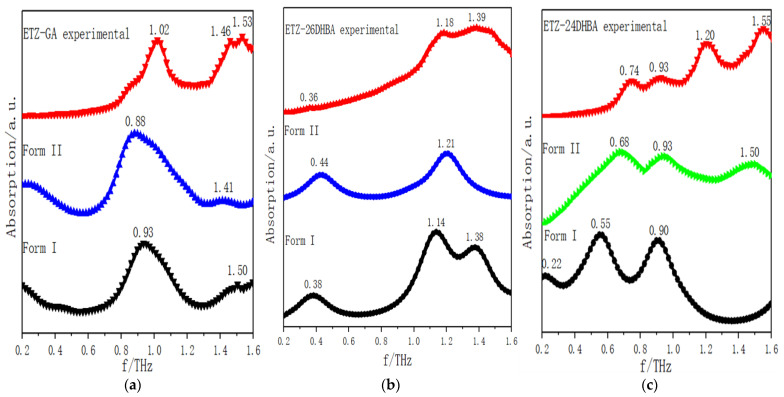
Comparisons of experimental and simulated THz spectra of (**a**) ETZ-GA cocrystal, (**b**) ETZ-26DHBA cocrystal and (**c**) ETZ-24DHBA cocrystal.

In order to focus on describing the primary hydrogen bond synthon displayed in the molecular structure, according to the structural information of ETZ with three CCFs and the synthetic law of hydrogen bond formation, we simulated two theoretically possible molecular structures of ETZ-GA, ETZ-26DHBA and ETZ-24DHBA cocrystals, respectively (as shown in Figure 2, Figure 3 and Figure 4). To easily visualize the hydrogen bonding effects among them, the intramolecular hydrogen bond interaction of ETZ is shown with a red shadow. Meanwhile, the intermolecular hydrogen bond interaction between ETZ and the three coformers is shown with a green shadow. Figure 6 shows comparisons of the experimental and simulated THz spectra of the ETZ-GA, ETZ-26DHBA and ETZ-24DHBA cocrystals. As shown in Figure 6a, for the ETZ-GA cocrystal, the two characteristic peaks at 0.93 and 1.50 THz of theoretical form I are close to those at 1.02 and 1.53 THz of the experimental spectrum. However, at 0.88 and 1.41 THz, theoretical form II also has two characteristic peaks that are relatively matched to the experimental spectrum at 1.02 and 1.46 THz. Thus, comparing the experimental and simulated THz spectral data of the ETZ-GA cocrystal in detail (Supporting Information Table S1), it is found that the two characteristic peaks of theoretical form I (0.93 THz: −8.82% and 1.50 THz: −1.96%) have lower error than theoretical form II (0.88 THz: −13.73% and 1.41 THz: −3.42%). At the same time, the total electronic energies of the two theoretical forms for the ETZ-GA cocrystal were calculated using DFT geometry optimization (Supporting Information Table S2). Theoretical form I has a lower total electronic energy (form I: −1756.6418 hartree) compared to theoretical form II (form II: −1756.6365 hartree), which indicates that the structure of theoretical form I is more stable. According to the above analysis, theoretical form I is more in line with the actual formation mode of the ETZ-GA cocrystal. However, the characteristic peaks at 0.93 and 1.50 THz of theoretical form I have a certain red shift compared with the experimental spectrum, and the characteristic peak at 1.46 THz of the experimental spectrum does not appear in theoretical form I of the ETZ-GA cocrystal. Notably, the primary hydrogen bond synthon in theoretical form I of the ETZ-GA cocrystal is consistent with that reported by Trivedi et al. [36]. Similarly, as shown in Figure 6b, for the ETZ-26DHBA cocrystal, the characteristic peak at 1.39 THz in the experimental spectrum does not appear in theoretical form II, but the characteristic peaks at 0.38, 1.14 and 1.39 THz of theoretical form I are close to the experimental spectrum of the 26DHBA cocrystal. Although the characteristic peaks at 1.14 and 1.38 THz of theoretical form I have a certain red shift compared with the experimental spectrum, the characteristic peaks at 0.38 THz have a certain blue shift. In addition, both the characteristic peak position error and total electronic energy of theoretical form I are much lower than those of theoretical form II (Supporting Information Tables S1 and S2). Therefore, theoretical form I is more in line with the actual formation mode of the ETZ-26DHBA cocrystal, and the acid–amide heterosynthon in theoretical form I of the ETZ-26DHBA cocrystal is the same as reported by Sarma et al. [37]. As shown in Figure 6c, for the ETZ-24DHBA cocrystal, compared with the spectrum result of theoretical form I, the three characteristic peaks at 0.68, 0.93 and 1.50 THz of theoretical form II are close to the experimental spectrum of the 24DHBA cocrystal. However, the characteristic peaks at 0.68 and 1.50 THz of theoretical form II have a certain red shift compared with the experimental spectrum, and the characteristic peak at 1.20 THz of the experimental spectrum does not appear in theoretical form II of the ETZ-24DHBA cocrystal. Moreover, theoretical form II is more favorable in terms of characteristic peak position error and total electronic energy compared with theoretical form I (Supporting Information Tables S1 and S2). Therefore, theoretical form II is more in line with the actual formation mode of the ETZ-24DHBA cocrystal, and the primary hydrogen bond synthon in theoretical form II of the ETZ-24DHBA cocrystal is basically consistent with those reported by Sarma et al. [38]. In general, the reason for the frequency shift in the characteristic peaks of the three cocrystals is that the simulated result is obtained under absolute zero, whereas the THz spectrum of the experimental result is obtained at room temperature [40]. At the same time, only one structural unit of the cocrystal molecular network is calculated in the theoretical results, while the entire solid-state cocrystal molecular network is obtained in the THz spectrum of the experimental results, which may also cause some frequency shifts.

Primary hydrogen bond synthon comparisons were performed for the ETZ-GA, ETZ-26DHBA and ETZ-24DHBA cocrystals. For the ETZ-GA cocrystal, theoretical form I is more in line with the actual formation mode of cocrystals, which indicates that the ETZ-GA cocrystal forms an O−H∙∙∙O hydrogen bond between the -OH of GA and oxygen of the amide group of the ETZ molecule, and it is also found that ETZ forms a dimer through a supramolecular amide–amide homosynthon. For the ETZ-26DHBA cocrystal, theoretical form I is more in line with the actual formation mode of cocrystals, which indicates that the ETZ-26DHBA cocrystal is formed through a powerful supramolecular acid–amide heterosynthon. For the ETZ-24DHBA cocrystal, theoretical form II is more in line with the actual formation mode of cocrystals, which indicates that the ETZ-24DHBA cocrystal interacts with the O−H∙∙∙O hydrogen bond formed by the 4-hydroxy group of 24DHBA and the oxygen of the amide group of the ETZ molecule. The supramolecular synthons in the three cocrystal structures are inconsistent, because the -COOH group, with strong donor–receptor affinity and adjacent acceptor multiple phenolic -OH groups, can obtain good intramolecular hydrogen bond interactions at the binding site, which may bring about a better drug curative effect. At the same time, the positions of -OH groups interacting with adjacent molecules may also affect the lipophilicity of polycompounds. For instance, the polar groups (-OH and -COOH) of 26DHBA are related to the hydrogen bonds, indicating that the lipophilicity of the ETZ-26DHBA cocrystal is enhanced, thereby improving the permeability of the drug. In addition, the mode of O−H∙∙∙O hydrogen bond connection in the ETZ-24DHBA cocrystal is similar to the reported ETZ-4HBA cocrystal [20]. The structural similarity of the two cocrystals shows that the 2-hydroxy group of 24DHBA forms an intramolecular hydrogen bond with the adjacent -COOH group of 24DHBA, while the 4-hydroxy group of 24DHBA forms an intermolecular hydrogen bond with the oxygen of the amide group of the ETZ molecule. This shows that the position of the hydroxyl group will have an important influence on the formation of supramolecular synthons. However, when the GA coformer has three hydroxyl groups, ETZ first forms a dimer through a supramolecular amide–amide homosynthon, and then interacts with the O−H∙∙∙O hydrogen bond formed by the -OH of GA and the oxygen of the amide group of the ETZ molecule, resulting in the stoichiometric ratio of the ETZ-GA cocrystal being 2:1. It can be seen that the number of hydroxyl groups may also affect the formation of supramolecular synthons. The analysis of the three cocrystal structures shows that the position and number of hydroxyl groups in the coformers direct the formation of specific supramolecular synthons.

Gaussian-View is a software program especially designed for Gaussian functions. Its major objective is to set up the input file of the Gaussian function, and then show the output computed result of the Gaussian function in graphical view. Through Gaussian-View software, we can easily observe the various vibrational modes of the theoretical cocrystals of ETZ-GA, ETZ-26DHBA and ETZ-24DHBA (Supporting Information Figures S1–S3), and allocate the modes of vibration as shown in Table 1. For the ETZ-GA cocrystal, the crystal vibration at 0.93 THz obtained by theoretical simulation can be attributed to the in-plane bending vibration of ETZ and GA. The simulated characteristic peak of 1.50 THz mainly comes from the in-plane bending vibration of GA, the out-of-plane bending vibration of ETZ and the out-of-plane rocking vibration of H37-N36-H38. For the ETZ-26DHBA cocrystal, the characteristic peak at 0.38 THz can be attributed to the out-of-plane rocking vibration of O11=C10-O12-H13 and H31-N30-H32. The characteristic peak at 1.14 THz can be attributed to the in-plane bending vibration of 26DHBA, the out-of-plane rocking vibration of H31-N30-N32 and the torsional vibration of O29=C28-N30-H31. The simulated characteristic peak of 1.38 THz mainly comes from the in-plane bending vibration of 26DHBA, the out-of-plane bending vibration of the ETZ ring and the out-of-plane rocking vibration of H31-N30-H32. For the ETZ-24DHBA cocrystal, the characteristic peak at 0.68 THz can be attributed to the in-plane bending vibration of ETZ and GA and the torsional vibration of O34=C33-O35-H36. The characteristic peak at 0.93 THz can be attributed to the in-plane bending vibration of ETZ and GA and the torsional vibration of O34-H38-O37 and O12-H40-O39. The characteristic peak at 1.50 THz may be attributed to the in-plane bending vibration of ETZ and 24DHBA. It can be observed by assigning modes of vibration that the hydrogen bond synthons of the three cocrystals play an important role in altering their molecular structures and vibration modes. The absorption mechanism of the THz spectrum mainly motivates intermolecular vibration, which may be utilized to test the hydrogen bond effect among various model molecules sensitively [27,28,29]. Therefore, the hydrogen bond effect with the starting components has an important influence on the formation of cocrystals between their two molecular structures, as determined by comparing the experimental THz spectrum and theoretical results, which leads to the cocrystal being different from that of the starting components for the modes of vibration.

### 3.2. Raman Spectral Characterization and Analysis of ETZ-GA, ETZ-26DHBA and ETZ-24DHBA Cocrystals

**Figure 7:**
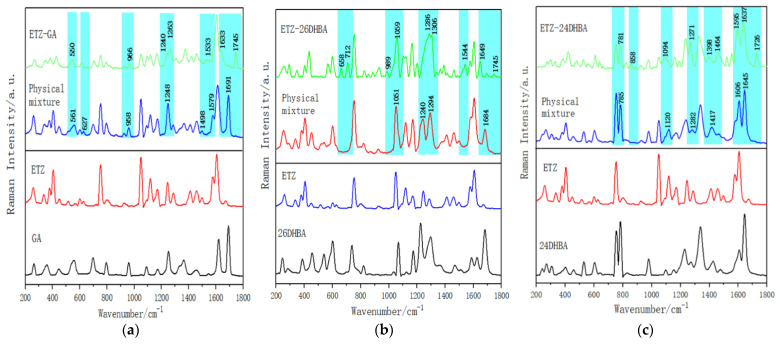
Experimental Raman spectra of (**a**) ETZ-GA cocrystal with ETZ, GA and physical mixture; (**b**) ETZ-26DHBA cocrystal with ETZ, 26DHBA and physical mixture; and (**c**) ETZ-24DHBA cocrystal with ETZ, 24DHBA and physical mixture in the wavenumber range of 200–1800 cm^−1^.

Raman spectroscopy has become one of the preferred methods to study the solid state of drugs over the past two decades due to its advantages of being fast, non-destructive and with no sample preparation required [26]. The Raman spectra of the single starting samples, corresponding physical mixtures and the cocrystals are shown in Figure 7. As shown in Figure 7a, some new characteristic peaks and frequency shifts could be observed in the ETZ-GA cocrystal by comparison with the Raman spectrum of the physical mixture. The Raman spectrum of the ETZ-GA cocrystal has unique characteristic peaks at 1533, 1633 and 1745 cm^−1^, and there are no corresponding characteristic peaks at 627 and 1248 cm^−1^ for the physical mixture. In addition, the Raman spectrum of the ETZ-GA cocrystal moves to a certain extent at the characteristic peaks of 550 and 966 cm^−1^. For the convenience of observation, the above characteristic peaks are covered by a light blue rectangular shadow in Figure 7a. Similarly, as shown in Figure 7b, the ETZ-26DHBA cocrystal has no corresponding characteristic peak in the Raman spectrum at 1684 cm^−1^, and new characteristic peaks are generated at 658, 712, 989 and 1544 cm^−1^ by comparison with the physical mixture. In addition, the ETZ-26DHBA cocrystal moves to a certain extent at the 1059, 1286 and 1306 cm^−1^ characteristic peaks of the Raman spectrum. As shown in Figure 7c, the ETZ-24DHBA cocrystal has no corresponding characteristic peak in the Raman spectrum at 1417 cm^−1^, and new characteristic peaks are generated at 858 and 1726 cm^−1^ by comparison with the physical mixture. Moreover, the ETZ-24DHBA cocrystal moves to a certain extent at the 781, 1094, 1271, 1606 and 1637 cm^−1^ characteristic peaks in the Raman spectrum. Through the analysis of the above Raman spectra, it is further proven that new substances are produced between ETZ and the three CCFs.

**Figure 8:**
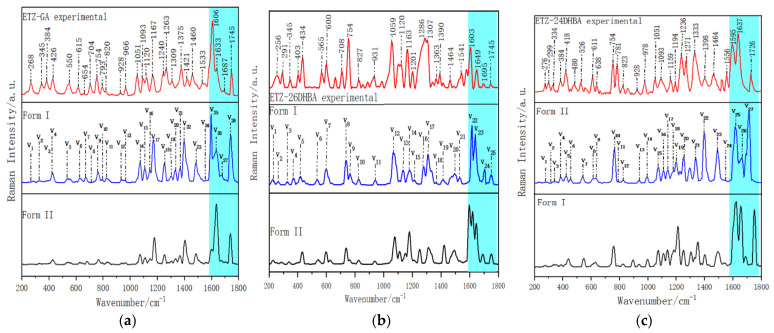
Comparisons of experimental and simulated Raman spectra of (**a**) ETZ-GA cocrystal, (**b**) ETZ-26DHBA cocrystal and (**c**) ETZ-24DHBA cocrystal.

Similarly, two possible theoretical forms were also simulated for the Raman spectra of ETZ-GA, ETZ-26DHBA and ETZ-24DHBA cocrystals (as shown in Figure 8). As shown in Figure 8a, for the ETZ-GA cocrystal, the four characteristic peaks of theoretical form I and the experimental Raman spectrum at 1606, 1633, 1687 and 1745 cm^−1^ are very consistent (as shown by the light blue shadow in Figure 8a) when comparing them with the Raman spectrum result of theoretical form II. In addition, the root mean square error (RMSE) of the overall characteristic peak position of theoretical form I (RMSE: 15.83) is less than that of theoretical form II (RMSE: 17.30), with the results shown in Table S3. RMSE can reflect the degree of difference between theoretical and experimental data, and the smaller the RMSE value, the more accurate the description of the experimental data by the theoretical data. Therefore, theoretical form I is more in line with the actual formation mode of the ETZ-GA cocrystal. As shown in Figure 8b, for the ETZ-26DHBA cocrystal, the four characteristic peaks of theoretical form I and the experimental Raman spectrum at 1603, 1649, 1695 and 1745 cm^−1^ are very consistent (as shown by the light blue shadow in Figure 8b) when comparing them with the Raman spectrum result of theoretical form II. Moreover, the RMSE of the overall characteristic peak position of theoretical form I (RMSE: 17.63) is smaller than that of theoretical form II (RMSE: 27.98), with the results shown in Table S4. Therefore, theoretical form I is more consistent with the actual formation mode of the ETZ-26DHBA cocrystal. As shown in Figure 8c, for the ETZ-24DHBA cocrystal, compared with the Raman spectrum result of theoretical form I, the three characteristic peaks of theoretical form II and the experimental Raman spectrum at 1595, 1637 and 1726 cm^−1^ are very consistent (as shown by the light blue shadow in Figure 8c). Moreover, the RMSE of the overall characteristic peak position of theoretical form II (RMSE: 15.98) is smaller than that of theoretical form I (RMSE: 30.73), with the results shown in Table S5. Therefore, theoretical form II is more in line with the actual formation mode of the ETZ-24DHBA cocrystal. In summary, the Raman theoretical results are in good agreement with the above THz theoretical results, and the Raman spectra also prove the formation of cocrystals and crystal formation modes. Through Gaussian-View software, we can also easily observe the various vibrational modes of the theoretical cocrystals of ETZ-GA, ETZ-26DHBA and ETZ-24DHBA, and allocate the modes of vibration (Supporting Information Tables S6–S8). According to the modes of vibration for ETZ-GA, ETZ-26DHBA and ETZ-24DHBA cocrystals in the table, it can be seen again that the hydrogen bond between the two starting components has an important influence in the corresponding cocrystallization process.

## 4. Conclusions

The cocrystals of ETZ with three CCFs (GA, 26DHBA and 24DHBA) were synthesized by the solvent evaporation method. ETZ, three CCFs (GA, 26DHBA and 24DHBA), corresponding physical mixtures and the corresponding cocrystals were characterized by vibrational spectra, such as THz and Raman spectroscopy. In addition, DFT was applied to optimize the possible theoretical molecular structures of the ETZ-GA, ETZ-26DHBA and ETZ-24DHBA cocrystals. The structural analysis showed that the position and number of hydroxyl groups in the coformers would have an important influence on the formation of specific supramolecular synthons. The ETZ-GA cocrystal interacted with the O−H∙∙∙O hydrogen bond formed by the -OH of GA and the oxygen of the amide group of the ETZ molecule, and it was also found that ETZ formed a dimer through a supramolecular amide–amide homosynthon. The ETZ-26DHBA cocrystal was formed by a powerful supramolecular acid–amide heterosynthon. The ETZ-24DHBA cocrystal interacted with the O−H∙∙∙O hydrogen bond formed by the 4-hydroxy group of 24DHBA and the oxygen of the amide group of the ETZ molecule. At the same time, according to the theoretical simulation results, we assigned the vibrational modes of ETZ-GA, ETZ-26DHBA and ETZ-24DHBA cocrystals, respectively, and found that they are closely related to the corresponding hydrogen bond, which further proves the bonding mode of hydrogen bonds and the significant effect of hydrogen bonds on cocrystal formation. THz and Raman spectroscopy are complementary to each other, which is very important for studying the modes of vibration and micro-molecular structures of the three cocrystals of ETZ and CCFs. It can be concluded that THz and Raman spectroscopy can well reveal the intermolecular and intramolecular hydrogen bonding interactions in the process of obtaining pharmaceutical cocrystals. The results offer an experimental and theoretical basis for studying the structures and vibrational spectra of pharmaceutical cocrystals.

## Figures and Tables

**Table 1:** Vibrational modes of the ETZ cocrystals in THz spectra.

Cocrystal	Experimental Resultsf/THz	Theoretical Resultsf/THz	Mode Assignment
ETZ-GA	1.02	0.93	In-plane bending vibration of two molecules of ETZ and one molecule of GA
	1.46	-	-
	1.53	1.50	In-plane bending vibration of GA, out-of-plane bending vibration of ETZ, out-of-plane rocking vibration of H37-N36-H38
ETZ-26DHBA	0.36	0.38	Out-of-plane rocking vibration of O11=C10-O12-H13 and H31-N30-H32
	1.18	1.14	In-plane bending vibration of 26DHBA, out-of-plane rocking vibration of H31-N30-H32, torsional vibration of O29=C28-N30-H31
	1.39	1.38	In-plane bending vibration of 26DHBA, out-of-plane bending vibration of the ring of ETZ, out-of-plane rocking vibration of H31-N30-H32
ETZ-24DHBA	0.74	0.68	Out-of-plane bending vibration of ETZ and 24DHBA, torsional vibration of O34=C33-O35-H36
	0.93	0.93	Out-of-plane bending vibration of ETZ and 24DHBA, torsional vibration of O34-H38-O37 and O12-H40-O39
	1.20	-	In-plane bending vibration of ETZ and 24DHBA
	1.55	1.50	In-plane bending vibration of ETZ and 24DHBA

## Data Availability

Not applicable.

## Supplementary Materials

The following supporting information can be downloaded at: https://www.mdpi.com/article/10.3390/ijms23158550/s1.
