# Supplementary material for: Pharmaceutical Cocrystals of Ethenzamide: Molecular Structure Analysis Based on Vibrational Spectra and DFT Calculations
Source: Int J Mol Sci. 2022 Aug 1;23(15):8550. doi: 10.3390/ijms23158550 (PMC9369141; doi:10.3390/ijms23158550)
Supplement: Supplementary file 1 [file ijms-23-08550-s001.zip › ijms-1836719-supplementary.pdf]

International Journal of
Molecular Sciences

MDPI

Supporting Information

Pharmaceutical Cocrystals of Ethenzamide: Molecular Structure Analysis Based on Vibrational Spectra and DFT Calculations

Mei Wan [1], Jiyuan Fang [1], Jiadan Xue [2], Jianjun Liu [1], Jianyuan Qin [1], Zhi Hong [1], Jiusheng Li [1] and Yong Du [1,*]

1 Centre for THz Research, China Jiliang University, Hangzhou 310018, China; wm1315286544@163.com (M.W.); jiyuanfang@foxmail.com (J.F.); jianjun@cjlu.edu.cn (J.L.); jyqin@cjlu.edu.cn (J.Q.); hongzhi@cjlu.edu.cn (Z.H.); lijsh@cjlu.edu.cn (J.L.)

2 Department of Chemistry, Zhejiang Sci-Tech University, Hangzhou 310018, China; jenniexue@126.com

* Correspondence: yongdu@cjlu.edu.cn; Tel.: +86-571-8687-5618

Table of Contents

Figure S1. Vibrational modes of ETZ-GA cocrystal at position of (a) 0.93 THz and (b) 1.50 THz. Page S2

Figure S2. Vibrational modes of ETZ-26DHBA cocrystal at position of (a) 0.38 THz, (b) 1.14 THz and (c) 1.38 THz. Page S3

Figure S3. Vibrational modes of ETZ-24DHBA cocrystal at position of (a) 0.68 THz, (b) 0.93 THz and (c) 1.50 THz. Page S4

Table S1. Comparisons of experimental and simulated THz spectral data of the ETZ cocrystals. Page S5

Table S2. Total electronic energies of the ETZ cocrystals from DFT structure simulations. Page S6

Table S3. Comparisons of experimental and simulated Raman spectral data of the ETZ-GA co-crystal. Page S7

Table S4. Comparisons of experimental and simulated Raman spectral data of the ETZ-26DHBA cocrystal. Page S8

Table S5. Comparisons of experimental and simulated Raman spectral data of the ETZ-24DHBA cocrystal. Page S9

Table S6. Vibrational modes for characteristic peaks of the ETZ-GA cocrystal in Raman spectra. Page S10

Table S7. Vibrational modes for characteristic peaks of the ETZ-26DHBA cocrystal in Raman spectra. Page S11

Table S8. Vibrational modes for characteristic peaks of the ETZ-24DHBA cocrystal in Raman spectra. Page S12

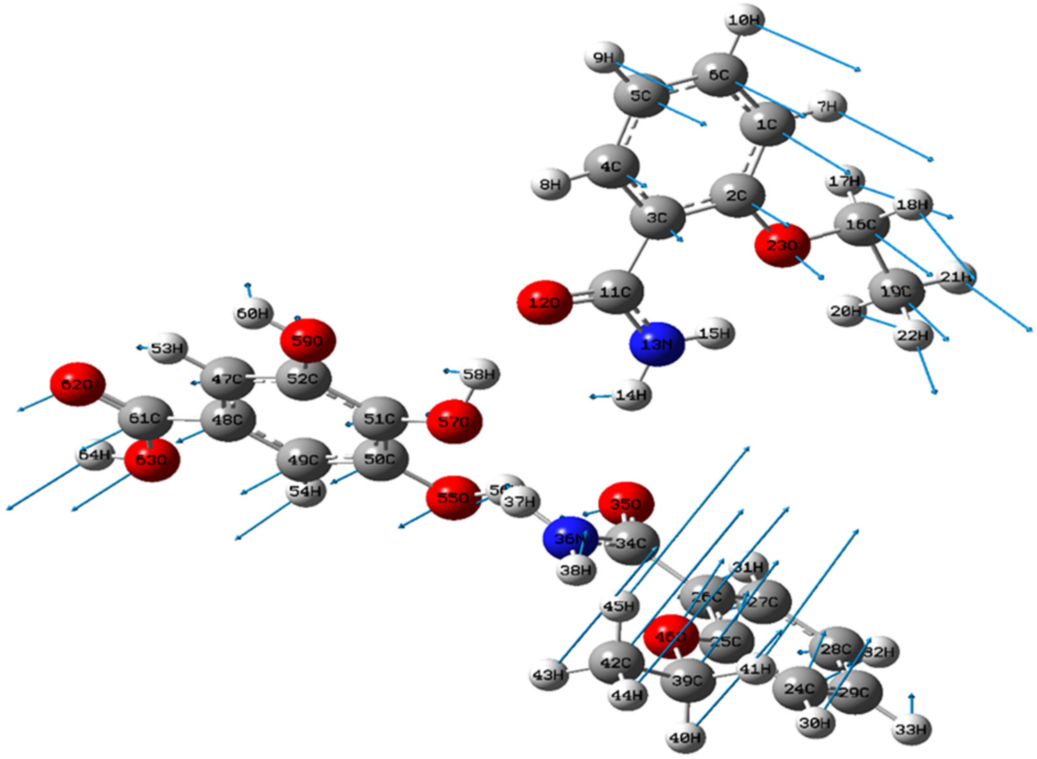

(**a**) 0.93 THz

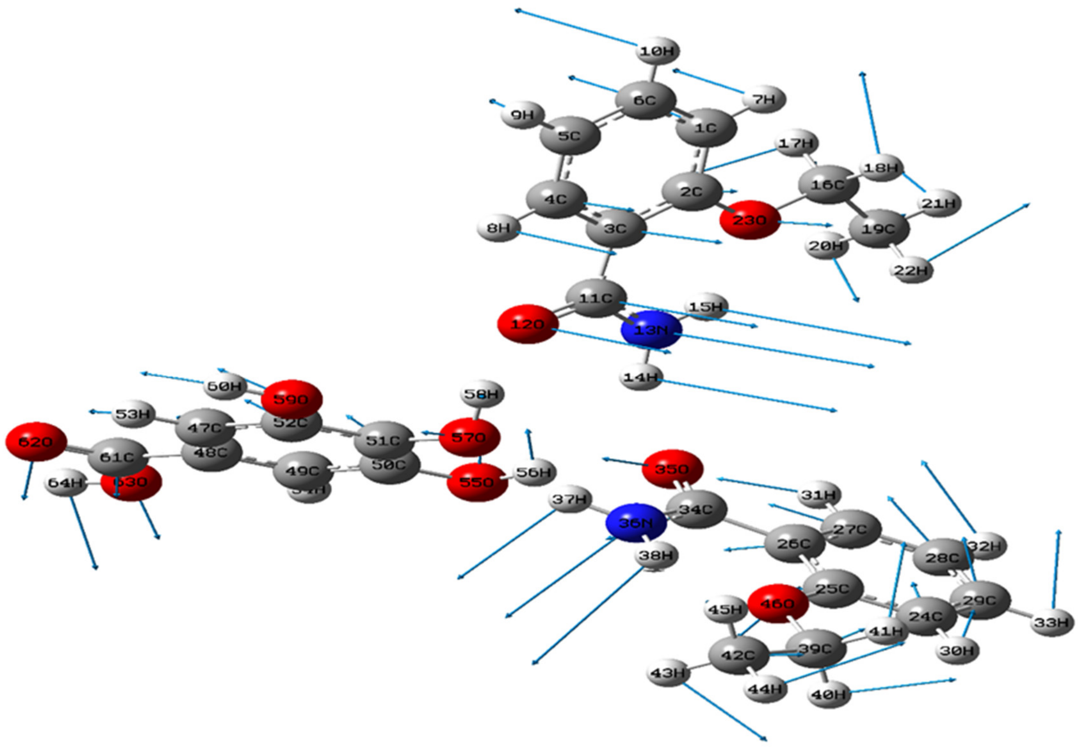

(**b**) 1.50 THz

Figure S1. Vibrational modes of ETZ-GA cocrystal at position of (**a**) 0.93 THz and (**b**) 1.50 THz.

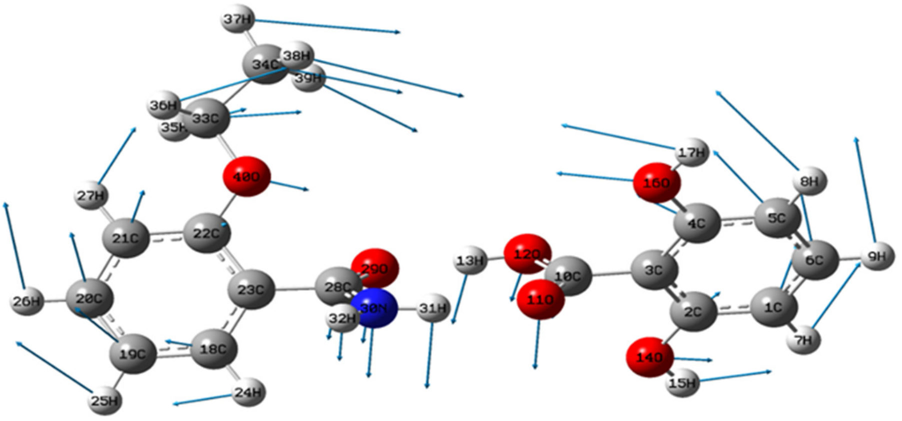

(**a**) 0.38 THz

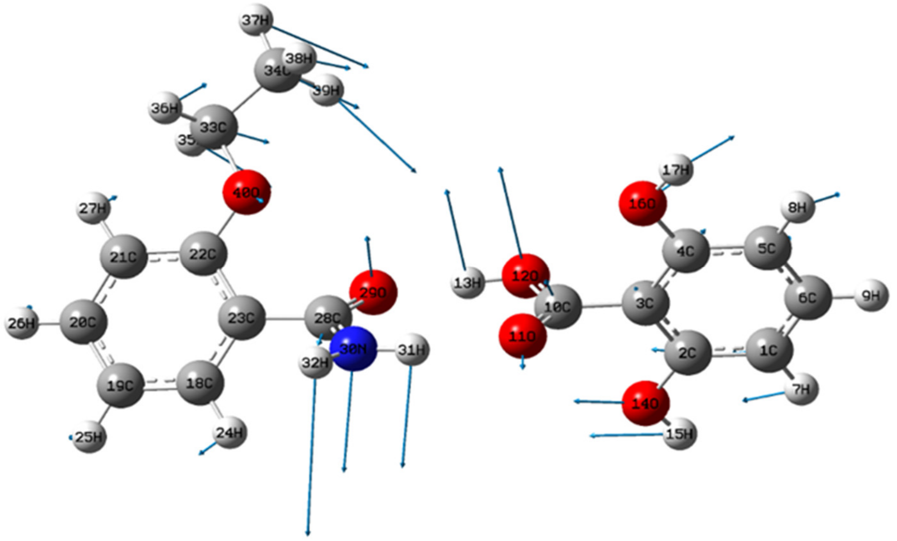

(**b**) 1.14 THz

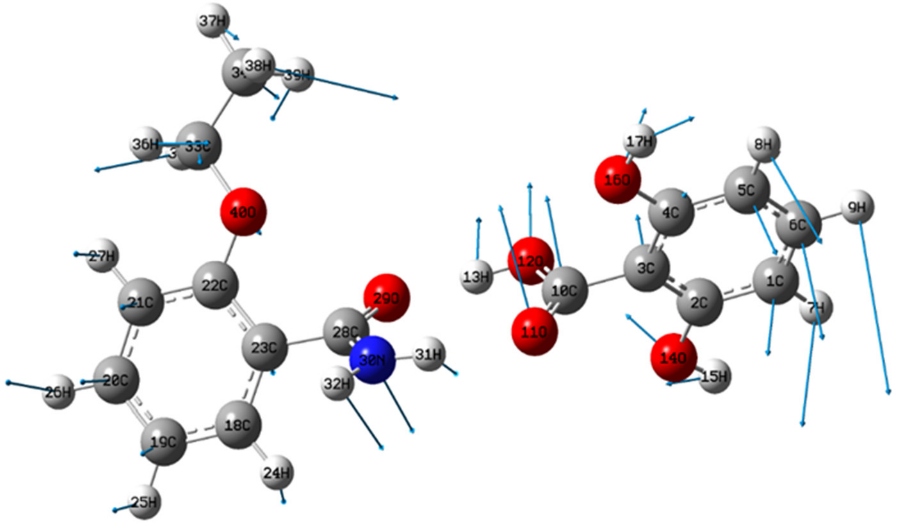

(**c**) 1.38 THz

Figure S2. Vibrational modes of ETZ-26DHBA cocrystal at position of (**a**) 0.38 THz, (**b**) 1.14 THz and (**c**) 1.38 THz.

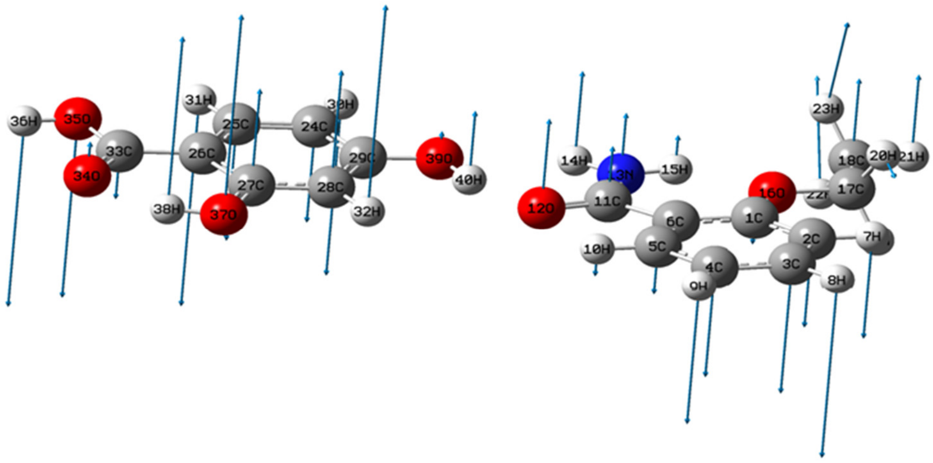

(**a**) 0.68 THz

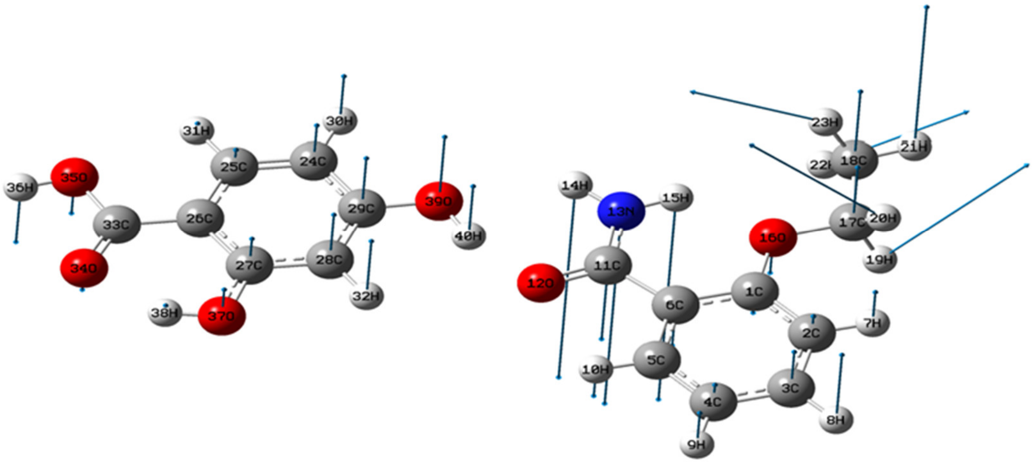

(**b**) 0.93 THz

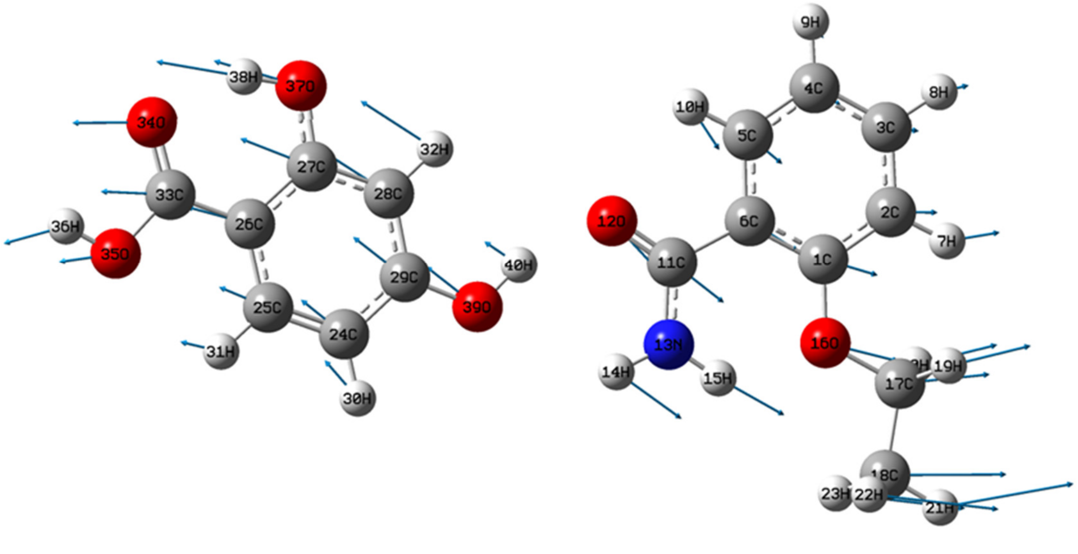

(**c**) 1.50 THz

Figure S3. Vibrational modes of ETZ-24DHBA cocrystal at position of (**a**) 0.68 THz, (**b**) 0.93 THz and (**c**) 1.50 THz.

Table S1. Comparisons of experimental and simulated THz spectral data of the ETZ cocrystals.

**Cocrystal**	**Experimental f/THz**	**Theoretical form I f/THz**	**Percent error**	**Theoretical form II f/THz**	**Percent error**
ETZ-GA	1.02	0.93	-8.82%	0.88	-13.73%
	1.46	—	—	1.41	-3.42%
	1.53	1.50	-1.96%	—	
ETZ-26DHBA	0.36	0.38	5.56%	0.44	22.22%
	1.18	1.14	-3.39%	1.21	2.54%
	1.39	1.38	-0.72%	—	—
ETZ-24DHBA	0.74	0.55	-25.68%	0.68	-8.11%
	0.93	0.90	-3.23%	0.93	0.00%
	1.20	—	—	—	—
	1.55	—	—	1.50	-3.23%

Table S2. Total electronic energies of the ETZ cocrystals from DFT structure simulations.

Cocrystal	Theoretical form	Total electronic energy (hartree)
ETZ-GA	form I	-1756.6418
	form II	-1756.6365
ETZ-26DHBA	form I	-1126.4120
	form II	-1266.4040
ETZ-24DHBA	form I	-1126.4155
	form II	-1126.4331

Table S3. Comparisons of experimental and simulated Raman spectral data of the ETZ-GA cocrystal.

Experimental Wavenumber/cm^{-1}	Theoretical Form I Wavenumber/cm^{-1}	Percent Error	Theoretical Form II Wavenumber/cm^{-1}	Percent Error
268	262	-2.24%	258	-3.73%
345	327	-5.22%	331	-4.06%
384	381	-0.78%	377	-1.82%
426	428	0.47%	428	0.47%
550	539	-2.00%	543	-1.27%
615	632	2.76%	628	2.11%
654	671	2.60%	671	2.60%
704	694	-1.42%	682	-3.13%
754	763	1.19%	767	1.72%
793	790	-0.38%	790	-0.38%
820	825	0.61%	833	1.59%
928	937	0.97%	941	1.40%
966	960	-0.62%	964	-0.21%
1051	1072	2.00%	1076	2.38%
1093	1106	1.19%	1110	1.56%
1120	1145	2.23%	1149	2.59%
1167	1172	0.43%	1176	0.77%
1240	1253	1.05%	1253	1.05%
1263	1299	2.85%	1303	3.17%
1309	1330	1.60%	1334	1.91%
1375	1365	-0.73%	1361	-1.02%
1421	1396	-1.76%	1403	-1.27%
1460	1484	1.64%	1488	1.92%
1533	1562	1.89%	1558	1.63%
1606	1596	-0.62%	1600	-0.37%
1633	1635	0.12%	1635	0.12%
1687	1673	-0.83%	1670	-1.01%
1745	1743	-0.11%	1739	-0.34%
	RMSE	15.83	RMSE	17.30

RMSE: root mean square error.

Table S4. Comparisons of experimental and simulated Raman spectral data of the ETZ-26DHBA cocrystal.

**Experimental Wavenumber/cm^{-1}**	**Theoretical Form I Wavenumber/cm^{-1}**	**Percent error**	**Theoretical form II wavenumber/cm^{-1}**	**Percent Error**
256	227	-11.33%	227	-11.33%
291	262	-9.97%	250	-14.09%
345	327	-5.22%	316	-8.41%
403	370	-8.19%	339	-15.88%
434	420	-3.23%	431	-0.69%
565	536	-5.13%	543	-3.89%
600	597	-0.50%	597	-0.50%
708	736	3.95%	736	3.95%
754	763	1.19%	763	1.19%
827	825	-0.24%	825	-0.24%
931	941	1.07%	937	0.64%
1059	1068	0.85%	1110	4.82%
1120	1133	1.16%	1153	2.95%
1163	1180	1.46%	1180	1.46%
1201	1214	1.08%	1211	0.83%
1286	1280	-0.47%	1249	-2.88%
1307	1307	0.00%	1311	0.31%
1363	1361	-0.15%	1361	-0.15%
1390	1415	1.80%	1423	2.37%
1464	1492	1.91%	1492	1.91%
1541	1530	-0.71%	1596	3.57%
1603	1616	0.81%	1619	1.00%
1649	1639	-0.61%	1646	-0.18%
1695	1704	0.53%	1689	-0.35%
1745	1751	0.34%	1751	0.34%
	RMSE	17.63	RMSE	27.98

RMSE: root mean square error.

Table S5. Comparisons of experimental and simulated Raman spectral data of the ETZ-24DHBA cocrystal.

Experimental wavenumber / cm^{-1}	Theoretical form I wavenumber / cm^{-1}	Percent error	Theoretical form II wavenumber / cm^{-1}	Percent error
276	277	0.36%	281	1.81%
299	308	3.01%	312	4.35%
334	335	0.30%	339	1.50%
384	385	0.26%	385	0.26%
418	416	-0.48%	420	0.48%
480	439	-8.54%	454	-5.42%
526	524	-0.38%	539	2.47%
611	547	-10.47%	617	0.98%
638	632	-0.94%	632	-0.94%
754	694	-7.96%	763	1.19%
781	759	-2.82%	786	0.64%
823	825	0.24%	825	0.24%
928	937	0.97%	937	0.97%
978	998	2.04%	995	1.74%
1051	1072	2.00%	1072	2.00%
1093	1110	1.56%	1110	1.56%
1159	1137	-1.90%	1141	-1.55%
1194	1211	1.42%	1180	-1.17%
1236	1249	1.05%	1199	-2.99%
1271	1303	2.52%	1249	-1.73%
1333	1353	1.50%	1338	0.38%
1398	1400	0.14%	1396	-0.14%
1464	1492	1.91%	1492	1.91%
1556	1623	4.31%	1546	-0.64%
1595	1654	3.70%	1616	1.32%
1637	1688	3.12%	1662	1.53%
1726	1750	1.39%	1712	-0.81%
	RMSE	30.73	RMSE	15.98

RMSE: root mean square error.

Table S6. Vibrational modes for characteristic peaks of the ETZ-GA cocrystal in Raman spectra.

Mode	Theoretical wavenumber / cm^{-1}	Experimental wavenumber / cm^{-1}	Mode assignment
ν_1	262	268(w)	ρ(H17-C16-H18, O12=C11-N13-H14)
ν_2	327	345(w)	δ(O62=C61-O63-H64), ρ(H37-N36-H38, H40-C39-H41)
ν_3	381	384(w)	Def(R2), ρ(H37-N36-H38, H40-C39-H41)
ν_4	428	426(m)	Def(R2), Def(R3), ρ(O62=C61-O63-H64)
ν_5	539	550(m)	ω(H15-N13-C11, H37-N36-H38), Def(R1)
ν_6	632	615(w)	ρ(H37-N36-H38, H14-N13-H15), Def(R2)
ν_7	671	654(m)	δ(O62=C61-O63-H64), Def(R3)
ν_8	694	704(m)	τ(H14-N13-H15), Def(R1)
ν_9	763	754(w)	Def(R2), ρ(H14-N13-H15, H37-N36-H38)
ν_{10}	790	793(s)	Def(R3), δ(O62=C61-O63-H64), ρ(H37-N36-H38)
ν_{11}	825	820(s)	ρ(H37-N36-H38, H40-C39-H41), Def(R2)
ν_{12}	937	928(w)	Def(R2), ρ(H37-N36-H38, H40-C39-H41)
ν_{13}	960	966(vw)	Def(R3), δ(O62=C61-O63-H64)
ν_{14}	1072	1051(m)	Def(R2)
ν_{15}	1106	1093(m)	Def(R1), ρ(H14-N13-H15)
ν_{16}	1145	1120(m)	ρ(H14-N13-H15, H37-N36-H38)
ν_{17}	1172	1167(m)	ρ(H37-N36-H38), Def(R2)
ν_{18}	1253	1240(w)	Def(R2), ρ(H37-N36-H38), θ(C25-O46)
ν_{19}	1299	1263(s)	Def(R1), ρ(H14-N13-H15, H37-N36-H38)
ν_{20}	1330	1309(s)	Def(R2)
ν_{21}	1365	1375(s)	δ(H37-N36-H38), Def(R3)
ν_{22}	1396	1421(w)	Def(R3), δ(O62=C61-O63-H64)
ν_{23}	1484	1460(m)	Def(R2), δ(H40-C39-H41)
ν_{24}	1562	1533(m)	Def(R3), ρ(O62=C61-O63-H64)
ν_{25}	1596	1606(s)	θ(C34=O35), Def(R2), δ(H14-N13-H15, H37-N36-H38)
ν_{26}	1635	1633(s)	θ(C11=O12), Def(R1), Def(R3), ρ(H37-N36-H38)
ν_{27}	1673	1687(m)	θ(C34=O35), δ(H14-N13-H15, H37-N36-H38)
ν_{28}	1743	1745(m)	θ(C61=O62), Def(R3)

Vw-very weak, w-weak, m-medium, s-strong, θ-stretching, ρ-in-plane rocking vibration, δ-scissor, ω-out of plane rocking vibration, τ-torsion, Def-Deformation.

Table S7. Vibrational modes for characteristic peaks of the ETZ-26DHBA cocrystal in Raman spectra.

Mode	Theoretical wavenumber / cm^{-1}	Experimental wavenumber / cm^{-1}	Mode assignment
ν_1	227	256(m)	τ(H31-N30-H32), ρ(O11=C10-O12-H13), ω(O29=C28-N30-H31)
ν_2	262	291(m)	ρ(H31-N30-H32, O11=C10-O12-H13, H35-C33-H36)
ν_3	327	345(w)	Def(R1), ρ(O11=C10-O12-H13, H35-C33-H36)
ν_4	370	403(m)	Def(R1), ρ(H31-N30-H32, H35-C33-H36)
ν_5	420	434(m)	ρ(O11=C10-O12-H13), Def(R2), δ(O34=C33-O35-H36, H26-C20=C21-H27)
ν_6	536	565(m)	ρ(O11=C10-O12-H13), Def(R2)
ν_7	597	600(m)	δ(O11=C10-O12-H13), Def(R2)
ν_8	736	708(m)	Def(R2), δ(O11=C10-O12-H13), ρ(H31-N30-H32)
ν_9	763	754(s)	Def(R1), ρ(H31-N30-H32)
ν_{10}	825	827(w)	Def(R1), δ(O11=C10-O12-H13), ρ(H31-N30-H32)
ν_{11}	941	931(w)	Def(R1), ρ(H35-C33-H36)
ν_{12}	1068	1059(s)	Def(R1)
ν_{13}	1133	1120(m)	ρ(H31-N30-H32, H35-C33-H36), δ(H24-C18=C19-H25)
ν_{14}	1180	1163(s)	δ(H7-C1=C6-H9, H8-C5=C6-H9)
ν_{15}	1214	1201(m)	Def(R2), θ(C10=O12)
ν_{16}	1280	1286(s)	θ(C22-O40), Def(R1)
ν_{17}	1307	1307(m)	ρ(H26-C20=C21-H27, H24-C18=C19-H25, H35-C33-H36)
ν_{18}	1361	1363(w)	Def(R2)
ν_{19}	1415	1390(w)	Def(R1), δ(H31-N30-C28)
ν_{20}	1492	1464(w)	θ(C2-O14, C4-O16), Def(R2)
ν_{21}	1530	1541(m)	δ(H35-C33-H36), Def(R1)
ν_{22}	1616	1603(s)	δ(H31-N30-H32), Def(R1), θ(C28=O29)
ν_{23}	1639	1649(m)	δ(H31-N30-H32), Def(R1)
ν_{24}	1704	1695(vw)	θ(C28=O29, C10=O11), δ(H31-N30-H32)
ν_{25}	1751	1745(vw)	θ(C28=O29, C10=O11), ρ(H31-N30-H32)

Vw-very weak, w-weak, m-medium, s-strong, θ-stretching, ρ-in-plane rocking vibration, δ-scissor, ω-out of plane rocking vibration, τ-torsion, Def-Deformation.

Table S8. Vibrational modes for characteristic peaks of the ETZ-24DHBA cocrystal in Raman spectra.

Mode	Theoretical wavenumber / cm^{-1}	Experimental wavenumber / cm^{-1}	Mode assignment
ν_1	281	276(w)	δ(C11-C6=C1, C33-C26=C1)
ν_2	312	299(w)	ρ(H15-N13-H14, H20-C17-H19, H23-C18-H22)
ν_3	339	334(w)	ρ(H20-C17-H19, H23-C18-H22), Def(R1), δ(C33-C26=C1)
ν_4	385	384(m)	δ(O34=C33-O35-H36, C11-C6=C5), Def(R1), ρ(H15-N13-H14, H23-C18-H22)
ν_5	420	418(m)	ρ(O34=C33-O35-H36, H20-C17-H19, H23-C18-H22), Def(R2)
ν_6	454	480(w)	ρ(O34=C33-O35-H36), δ(C11-C6=C5), Def(R1)
ν_7	539	526(m)	ρ(H15-N13-H14), δ(O34=C33-O35-H36), Def(R1)
ν_8	617	611(m)	ρ(C11-C6=C1), δ(O34=C33-O35-H36), Def(R1),
ν_9	632	638(w)	Def(R2), ρ(H15-N13-H14)
ν_{10}	763	754(s)	Def(R1), δ(O34=C33-O35-H36)
ν_{11}	786	781(s)	ω(H8-C3=C2-H7, O12=C11-C6), τ(H15-N13-C11)
ν_{12}	825	823(w)	ρ(H15-N13-H14), Def(R2)
ν_{13}	937	928(vw)	Def(R2)
ν_{14}	995	978(m)	Def(R1)
ν_{15}	1072	1051(m)	Def(R2)
ν_{16}	1110	1093(m)	ρ(H15-N13-H14), Def(R2)
ν_{17}	1141	1159(m)	ρ(H15-N13-H14)
ν_{18}	1180	1194(w)	ω(O16-C17-C18), ρ(H20-C17-H19)
ν_{19}	1199	1236(s)	ρ(H31-C25=C24-H30, C33-C26=C27)
ν_{20}	1249	1271(s)	θ(C1-O16), Def(R2), δ(H8-C3=C4-H9)
ν_{21}	1338	1333(s)	ρ(H31-C25=C24-H30), δ(C33-C26=C27), Def(R1)
ν_{22}	1396	1398(w)	Def(R1), δ(C11-N13-H14), ρ(H8-C3=C4-H9, H31-C25=C24-H30)
ν_{23}	1492	1464(m)	θ(C27-O37), ρ(H8-C3=C4-H9), Def(R1)
ν_{24}	1546	1556(m)	Def(R1), ρ(H31-C25=C24-H30), θ(C27=C26)
ν_{25}	1616	1595(s)	Def(R2), δ(H15-N13-H14)
ν_{26}	1662	1637(s)	θ(C25=C24, C28=C27, C33=O34), Def(R1)
ν_{27}	1712	1726(m)	θ(C33=O34, C11=O12), δ(H15-N13-H14)

Vw-very weak, w-weak, m-medium, s-strong, θ-stretching, ρ-in-plane rocking vibration, δ-scissor, ω-out of plane rocking vibration, τ-torsion, Def-Deformation.
